# The Ecological Roles of Medium and Small Carnivores in the Terrestrial Animal Community in Liancheng National Nature Reserve, China

**DOI:** 10.3390/ani12243518

**Published:** 2022-12-13

**Authors:** Tengwei Su, Qian Li, Xiaojuan Wang, Guofa Cui, Zihong Man, Wentao Li, Minyan Zhao

**Affiliations:** 1 School of Ecology and Nature Conservation, Beijing Forestry University, Beijing 100083, China; 2 School of Ecological and Environmental Engineering, Yunnan Forestry Technological College, Kunming 650224, China; 3 School of Geography and Ecotourism, Southwest Forestry University, Kunming 650224, China; 4 Administration Bureau of Liancheng National Nature Reserve of Gansu Province, Lanzhou 730399, China; 5 Institute of Tibetan Plateau Research, Chinese Academy of Sciences, Beijing 100101, China

**Keywords:** medium and small carnivores, spatial association, network analysis, camera trapping, Liancheng National Nature Reserve

## Abstract

**Simple Summary:**

Much less attention has been paid to medium and small carnivores than to large ones, leading to a shortfall of knowledge regarding their ecological roles. It is particularly necessary to improve the research on the ecological roles of medium and small carnivores in the context of the massive decline in the number of large carnivores around the world. For this purpose, a study on the ecological roles of medium and small carnivores was conducted in Liancheng Reserve, China. On the basis of constructing a spatial association network of species, we analyzed the status of medium and small carnivores in the network, characterized their association, and assessed their contribution to the maintenance of the community in the absence of large carnivores, such as gray wolf (*Canis lupus*) and brown bear (*Ursus arctos*). Furthermore, the development trends of the community were predicted in order to act as a guide for the direction and focus of conservation efforts.

**Abstract:**

It is vitally important to understand the ecological roles of medium and small carnivores in the context of the massive decline in the number of large carnivores around the world. Based on a spatial association network of terrestrial birds and mammals, this study analyzed the ecological roles of medium and small carnivores in the community in Liancheng National Nature Reserve. From October 2019 to June 2020, we obtained 3559 independent detections of 20 terrestrial birds and mammals from 112 camera traps. There are seven species that are medium and small carnivores present in the study area, including red fox (*Vulpes vulpes*), leopard cat (*Prionailurus bengalensis*), Chinese mountain cat (*Felis bieti*), stone marten (*Martes foina*), Asian badger (*Meles leucurus*), Siberian weasel (*Mustela sibirica*) and mountain weasel (*Mustela altaica*). By calculating the Phi coefficient of all species pairs, a spatial association network composed of twelve species was constructed. We analyzed the characterization of spatial associations by the Shannon–Wiener index and Lambda statistic. The results showed that: (1) the status of the network reflects the changes of community composition and structure after the decline in large carnivores and other species; (2) with the exception of the Chinese mountain cat and stone marten, the other five medium and small carnivores were located in the network, which played an important role in the complexity of the network and the maintenance of the community; (3) the medium and small carnivores could not take the place of the large carnivores in order to control the population of herbivores, such as Siberian roe deer (*Capreolus pygargus*) and Himalayan marmot (*Marmota himalayana*). The results of this study provide guidance for determining the direction and focus of conservation efforts.

## 1. Introduction

Large carnivores, such as the Serengeti lions (*Panthera leo*) [1], snow leopard (*P. uncia*) [2] and gray wolf *(Canis lupuse*) [3], often play the role of the apex predator in the ecosystem. They can control the population of large herbivores and are important for maintaining the health of the ecosystem [4,5,6]. As a result, large carnivores receive more attention than medium and small carnivores. Large carnivores usually require large prey and expansive habitats, and illegal hunting and habitat fragmentation have led to their decline and extinction worldwide [7]. In this context, we must raise the following questions: What will happen to the communities of animals in these areas? Will mesopredator release occur [8]? Can medium and small carnivores partially fulfil the ecological roles of large carnivores? At present, there are few studies on these issues. Thus, we must overcome the shortfalls of the ecological role of medium and small carnivores [9,10].

An ecological community is a network of species connected by ecological relationships. Species survive and evolve based on networks [11]. Based on the structure of a network, we can understand the position of the species in the community and how important they are to maintaining the stability of the network [12]. Interspecific interactions occur in a certain space and time, and interspecific relationships can be detected through spatial overlap [13]. Today, camera-trapping has been widely used in the investigation of terrestrial birds and mammals, which can enable the collection of a lot of spatial distribution data about species over a long period and across a wide geographical range, representing an effective tool for the study of interspecific association. From 2018 to 2021, Yang et al. [14], Zhou et al. [15], Li et al. [16] and Liu et al. [17] studied the spatial association of terrestrial birds and mammals by camera-trapping in the Wolong and Tangjiahe National Nature Reserves, Sichuan, China. Based on the “presence–absence” data of species, the phi coefficient and chi-square test were used to identify the species pairs with significant positive spatial association. Then, a spatial association network, which included multiple species, was constructed to realize the quantification and visualization of the integrated species association.

The Liancheng National Nature Reserve (LNNR) is located in Yongdeng County, Lanzhou City, Gansu Province, China, which is the ecotone of the Loess Plateau and the Qinghai-Tibet Plateau. Brown bear (*Ursus arctos*), gray wolf, Eurasian lynx (*Lynx lynx)* and other large carnivores used to be distributed here. However, these species have been declining or have disappeared from the reserve due to human disturbances, such as commercial logging, grazing and hunting. Despite a current 20-year ban on logging and hunting, large carnivores have shown no signs of recovery, while populations of species such as the red fox (*Vulpes vulpes*), Asian badger (*Meles leucurus*), Siberian roe deer (*Capreolus pygargus*), Himalayan marmot (*Marmota himalayana*) and blue eared pheasant (*Crossoptilon auritum*) have risen markedly [18]. In the absence of large predators, what is the community like in Liancheng Reserve? What roles do medium and small carnivores play in the community? Finding the answers to these questions will be helpful for conservation and wildlife management efforts.

In this study, the spatial distribution data of terrestrial birds and mammals in the LNNR were collected by camera-trapping, and the objectives were as follows: (1) to construct a spatial association network of terrestrial birds and mammals; (2) to understand the status and the ecological roles of medium and small carnivores in the community; and (3) to predict the trends of community development.

## 2. Materials and Methods

### 2.1. Study Area

**Figure 1:**
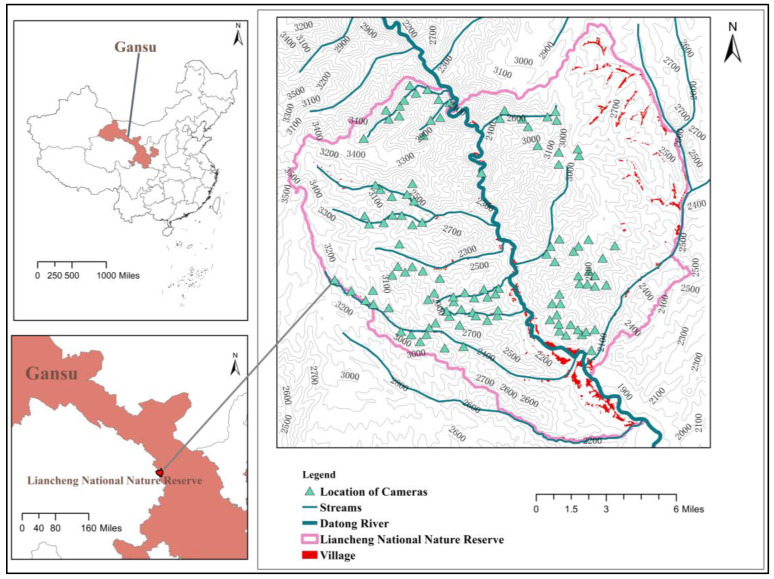
Location of LNNR and camera traps.

The LNNR(102°36′–102°55′ E,36°33′–36°48′ N, Figure 1) is located in the transition zone between the northeastern Qinghai-Tibet Plateau and western Loess Plateau. The reserve was established in 2001 and covered a total area of 479.3 km^2^. The elevation ranges from 1870 to 3616 m. The region has a typical temperate continental climate with a mean annual temperature of 7.4 °C; a mean annual precipitation of 419 mm, which is mostly concentrated between June and September every year, with occasional snowfall in the winter; and a mean annual evaporation level of 1542 mm. The Datong River runs through the reserve from north to south, with numerous ravines distributed on the river sides. The reserve preserves a large area of primeval Qinghai spruce (*Picea crassifolia*) forest and Sabina chinensis (*Sabina przewalskii*) forest and distributes seven types of vegetation forms, including deciduous broad-leaved forest, temperate coniferous forest, temperate coniferous and broad-leaved forest, cold-temperature coniferous forest, cold-temperature coniferous and broad-leaved forest, deciduous broad-leaved shrubland and temperate grass and weed meadow. A variety of vegetation types contribute to the multiple biotope and abundant animal species. According to a comprehensive scientific survey completed in 2018 (unpublished), there are 23 orders, 60 families and 190 terrestrial vertebrates, among them there are 5 orders, 16 families, 33 mammalian species and 16 orders, 37 families, 148 bird species including 9 Galliformes. According to the IUCN Red List, the endangered species include Dhole (*Cuon alpinus*), alpine musk deer (*Moschus chrysogaster*), forest musk deer (*M. berezovskii*) and saker falcon (*Falco cherrug*); vulnerable species include the Chinese mountain cat (*Felis bieti*) and Eastern imperial eagle (*Aquila heliaca*); and near-threatened species include the Eurasian otter (*Lutra lutra*), Chinese grouse (*Bonasa sewerzowi*) and cinereous vulture (*Aegypius monachus*) [18].

### 2.2. Data Collection

The data were collected using camera traps in the LNNR from October 2019 to June 2020. The reserve was divided into 1 km × 1 km grids using ArcGIS Desktop 10.7 (Esri Inc., Redlands, CA, USA). After avoiding villages, roads, farmland, grazing and inaccessible areas, camera traps were installed along the ravines. One camera trap was placed in each kilometer grid, where it was as close as possible to the beast tracks and water and no attractant was used. The distance between the camera traps was >300 m. A total of 130 camera traps were deployed (Figure 1). The cameras captured 3 consecutive photos, followed by 10 s of video recording (active 24 h a day). Every camera was fixed to a tree trunk 50–80 cm above the ground with the lens pointing towards the horizon. Shrubs and debris 1–4 m in front of the camera were removed to capture semi-terrestrial species in the long shot (on trees) and in medium and close shots. The battery and storage card were replaced every 3 months. After obtained the field data, a dataset was established for each camera. Then, we checked the shots in the storage card one by one to identify the species in the videos and pictures, and counted the number of detected traps (DTS) and independent detections [19] (IDS), analyzed the distribution of vegetation types (VTS) and elevation range (ER)/elevation difference (ED) for each species. Due to the difficulty of individual identification accuracy, this study only extracted the “presence–absence” binary data for each species from each camera.

### 2.3. Data Analysis

#### 2.3.1. Analysis of Species Spatial Associations

Based on the binary data, the phi coefficient was used to measure the spatial associations between species. The phi coefficient was calculated, then the significance level was subsequently determined [20]. First, a 2 × 2 contingency table was created (Table 1), where A, B, C and D denote the number of camera traps in four scenarios: (1) both species X and Y were recorded; (2) X was not recorded but Y was recorded; (3) X was recorded but Y was not recorded; and (4) both species X and Y was not recorded. *N* = A + B + C + D is the total number of camera traps (*N* = 112).

The data were entered into the following equation:

(1) $$r_{\varphi }=\frac{\left|\mathrm{AD}\;-\;\mathrm{BC}\right|}{\sqrt{\left(A\;+\;B\right)\left(C\;+\;D\right)\left(A\;+\;C\right)\left(B\;+\;D\right)}}$$

where $r_{\varphi }$ is the phi coefficient and indicates the degree of interspecific associations. (AD − BC) > 0 is a positive association and (AD − BC) < 0 is a negative association. $r_{\varphi }$ ranges from 0–1; the closer the value is to 1, the stronger the association.

To exclude chance associations caused by random factors, a chi-squared test was performed on the calculated phi coefficients using the following equation:

(2) $$\chi^{2}=\frac{N{\left(\left|\mathrm{AD}\;-\;\mathrm{BC}\right|-\frac{N}{2}\right)}^{2}}{\left(A\;+\;B\right)\left(C\;+\;D\right)\left(A\;+\;C\right)\left(B\;+\;D\right)}$$

where A, B, C, D and *N* are the same as in formula 1 and χ^2^ is the chi-squared test. χ^2^ ≥ 3.84 (*p* ≤ 0. 05) indicates that $r_{\varphi }$ is significant, the presence of an ecology-based spatial association between two species and that the value is valid for further analysis. χ^2^ < 3.84 (*p* > 0.05) indicates that $r_{\varphi }$ is insignificant and the absence of an ecology-based spatial association between two species; the spatial association is caused by random factors and the value is invalid for further analysis.

Based on the phi coefficients, species pairs with significant positive associations were selected and the species were used as nodes to construct a network using spatial association analysis software, Netdraw V.2.148 (Analytic Technologies, Lexington, KY, USA).

#### 2.3.2. Characterization of Species Spatial Associations

Since the interaction between species pairs may present symmetric differences, the lambda statistic was used to test the asymmetry of each species pair. In a species pair, one species may benefit from another species and base its distribution on the presence of the other species, resulting in an asymmetric spatial association. This method measures the proportional reduction in error when predicting the presence of one species with the presence of another (i.e., the predictability of the occurrence of one species with the occurrence of another) [20]. The following calculation was performed based on the contingency table:

(3) $$L_{B}=\frac{\sum_{j=1}^{k}n_{Mj}-max\left(R_{i}\right)}{N-max\left(R_{i}\right)}$$

where $n_{Mj}$ is the maximum frequency of the *j*th column (when *j* = 1 (the column with the presence of species X), $n_{Mj}$ is larger than A and C, and when *j* = 2 (the column with the absence of species X), $n_{Mj}$ is larger than B and D), $max\left(R_{i}\right)$ is the maximum sum of rows (if (A + B) > (C + D), then $max\left(R_{i}\right)=\left(A+B\right)$; if (A + B) < (C + D), then $max\left(R_{i}\right)=\left(C\;+D\right)$; *N* = A + B + C + D), and $L_{B}$ is the upper limit of the predictability of species B (Y in the contingency table) by species A (X in the contingency table).

The significance of $L_{B}$ was tested in two steps. The first step required the calculation of the variability of $L_{B}$ using the following equation:

(4) $$var\left(L_{B}\right)=\frac{\left(N-\sum_{j=1}^{k}n_{Mj}\right)\;\left(\sum_{j=1}^{k}n_{Mj}+max\left(R_{i}\right)-2\sum_{}^{\prime }n_{Mj}\right)}{{\left[N-max\left(R_{i}\right)\right]}^{3}}$$

where $\sum_{}^{\prime }n_{Mj}$ is the sum of the maximum column frequencies in the row of $max\left(R_{i}\right)$; $\sum_{}^{\prime }n_{Mj}\;$= $n_{Mj}$ as there was only one maximum column frequency in each row. The second step was the calculation of *z*-scores using the following equation:

(5) $$z=\frac{L_{B\;}-\lambda_{B0}}{\sqrt{var\left(L_{B}\right)}}$$

where $\lambda_{B0}$ is the lower limit of the predictability of species B by species A; the value was selected by the investigator. The *z*-scores were used to discover the *p*-values in a standard normal table, where *p* ≤ 0.05 indicated significant differences. *Z*-scores corresponding to *p* = 0.05 (*z*_*p* = 0.05_) were obtained and $\lambda_{B0}$ was calculated using the following equation:

(6) $$\lambda_{B0}=L_{B}-z_{p=0.05}\times \sqrt{var\left(L_{B}\right)}$$

where $\lambda_{B0}\leq 0$ indicates no predictability of species A for species B. For each species pair, the predictability of X for Y (and Y for X) was calculated. The predictability of X for Y was not necessarily equal to the predictability of Y for X. When two species predict each other, a bidirectional asymmetric association is formed, reflecting that two species tend to distribute in the space where the other appears and benefit from each other’s existence, which is a mutually benefiting relationship. When only one species is predictive of another species, a unidirectional asymmetric association is formed, reflecting that one species tends to distribute in the space where the other species appears. This tendency is derived from the former species benefiting from the existence of the latter species, which is a biasedly benefiting relationship. When there is no predictability between two species, a symmetrical association is formed, reflecting the needs of two species for common ecological resources (such as food resources and activity places) in the same space. The ecological relationship between two species has equal interests, which is a competitive relationship.

#### 2.3.3. Species Contribution to Spatial Network Complexity

The stability of a network is closely related to the complexity of the structure of the network [12]. The contributions of species to the complexity of the network are different. In this study, the Shannon–Wiener index was used to measure the diversity of interspecific associations, and the formula is as follows:

(7) $$H^{\prime }=-\sum_{i=1}^{S}P_{i}\times \ln(P_{i})$$

where *H*′ is the Shannon–Weiner index; *S* is the total number of positively associated species pairs; and *P_i_* is the proportion of association coefficients of species pair *I* relative to the total association coefficients of all species pairs. The larger the *H*′ value, the more complex the ecological relationship of a species in the community. The *H*′ values of all species were summed and averaged. When *H*′ of a species was less than the average value, the species was defined as a peripheral species, which indicated that it was less important for network maintenance. When *H*′ was greater than the average, the species was defined as a core species that considerably contributed to community maintenance, which is calculated as follows:

(8) $$C_{i}=\frac{H_{i}^{\prime }}{\sum_{i\;=1}^{S}H_{i}^{\prime }}\times 100\%$$

where *S* is the number of species; $H_{i}^{\prime }$ is the Shannon–Wiener index of the species *i*; $\overset{S}{\underset{i=1}{\sum }}H_{i}^{\prime }$ is the sum of Shannon–Wiener indices of all species; and *C_i_* is the contribution of species *i* to network maintenance. The greater the *C_i_*, the greater the contribution of species *i* and the greater its importance.

## 3. Results

### 3.1. Diversity of Species

After removing the camera traps that failed to work continuously due to the loss and malfunction, 3559 independent detections were collected from 112 camera traps from 1 October 2019 to 18 June 2020. The camera traps worked for a total of 29,232 trap days. A total of 5 orders, 8 families and 20 species of terrestrial birds and mammals were identified in the photos and videos (Table 2). A total of eight species of carnivores were detected. Except for the large carnivore Dhole, the others were medium and small carnivores, including the leopard cat, Chinese mountain cat, Asian badger, red fox, Siberian weasel, stone marten (*Martes foina*) and mountain weasel (*Mustela altaica*). There were three species of Cetartiodactyla, including sika deer (*Cervus nippon*), red deer (*C. elaphus*) and Siberian roe deer (*Capreolus pygargus*). There were two species of Lagomorpha, namely, Chinese red pika (*Ochotona erythrotis*) and woolly hare (*Lepus oiostolus*); there were two species in the order Rodentia, namely, Siberian chipmunk (*Tamias sibiricus*) and Himalayan marmot. There were five species of pheasants in the order Galliformes: Chinese grouse (*Tetrastes sewerzowi*), blue eared pheasant, blood pheasant (*Ithaginis cruentus*), chestnut-throated partridge (*Tetraophasis obscurus*) and common pheasant (*Phasianus colchicus*). The Muridae and Dipodidae species could not be identified. In view of their similar habits and ecological functions, they were combined into a special species “Muridae and Dipodidae” to perform the statistical analysis. Brown bear, gray wolf, Eurasian lynx, Pallas’s cat (*Otocolobus manul*), bharal (*Pseudois nayaur*) and alpine musk deer were not photographed in this survey.

**Figure 2:**
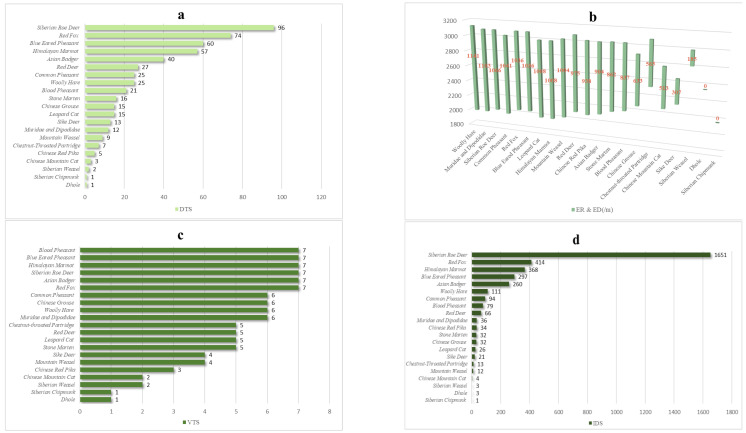
(**a**). The number of detected traps of species (DTS); (**b**) Distribution of elevation range (ER)/elevation difference (ED) of species (the red numbers indicate elevation difference); (**c**) Distribution of vegetation types of species (VTS); (**d**) The Number of independent detections of species (IDS).

It can be seen from the number of detected traps of species in Figure 2a, that the species whose detected traps number more than 30% of the total camera traps are Siberian roe deer (DTS = 96), red fox (DTS = 74), blue eared pheasant (DTS = 60), Himalayan marmot (DTS = 57) and Asian badger (DTS = 40), and those whose detected traps number less than 5% of the total camera traps are Dhole (DTS = 1), Siberian chipmunk (DTS = 1), Siberian weasel (DTS = 2), Chinese mountain cat (DTS = 3) and Chinese red pika (DTS = 5).

The distribution of elevation range of species can be seen in Figure 2b, the species whose distribution of elevation difference more than 1000 m are woolly hare (ED = 1141), Muridae and Dipodidae (ED = 1103), Siberian roe deer (ED = 1066), common pheasant (ED = 1041), red fox (ED = 1036), blue eared pheasant (ED = 1036), leopard cat (ED = 1008), Himalayan marmot (ED = 1008) and mountain weasel (ED =1 004). The distribution of elevation difference less than 600 m are chestnut-throated partridge (ED = 565), Chinese mountain cat (ED = 513), sika deer (ED = 307), Siberian Weasel (ED = 185), Dhole (ED = 0) and Siberian Chipmunk (ED = 0).

The distribution of vegetation types of species in Figure 2c shows that blood pheasant (VTS = 7), blue eared pheasant (VTS = 7), Himalayan marmot (VTS = 7), Siberian roe deer (VTS = 7), Asian badger (VTS = 7) and red fox (VTS = 7) were distributed in all types of vegetation in the reserve, and Dhole (VTS = 1), Siberian chipmunk (VTS = 1), Siberian weasel (VTS = 2) and Chinese mountain cat (VTS = 2) are only distributed in one–two types of vegetation in the reserve.

The number of independent detections of species in Figure 2d shows that the top five species of independent detections are Siberian roe deer (IDS = 1651), red fox (IDS = 414), Himalayan marmot (IDS = 368), blue eared pheasant (IDS = 297) and Asian badger (IDS = 260), and the number of independent detections less than 10 are Siberian chipmunk (IDS = 1), Dhole (IDS = 3), Siberian weasel (IDS = 3) and Chinese mountain cat (IDS = 4).

### 3.2. Spatial Associations of Medium and Small Carnivores

**Figure 3:**
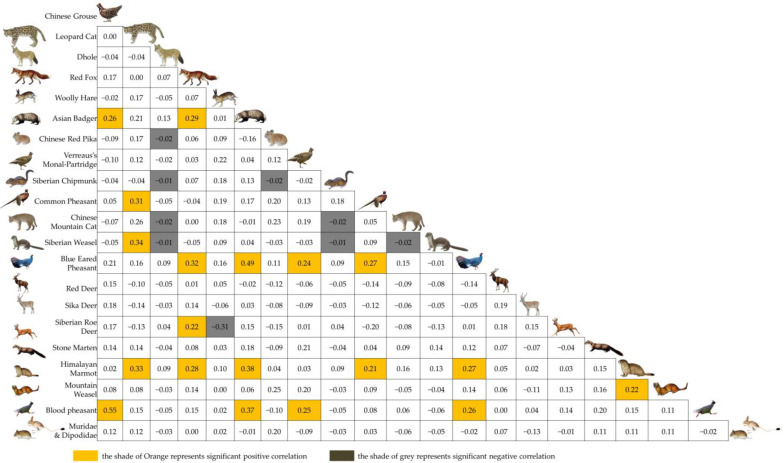
Semi-matrix graph of spatial association intensity (*r_φ_*) of terrestrial birds and mammals.

**Figure 4:**
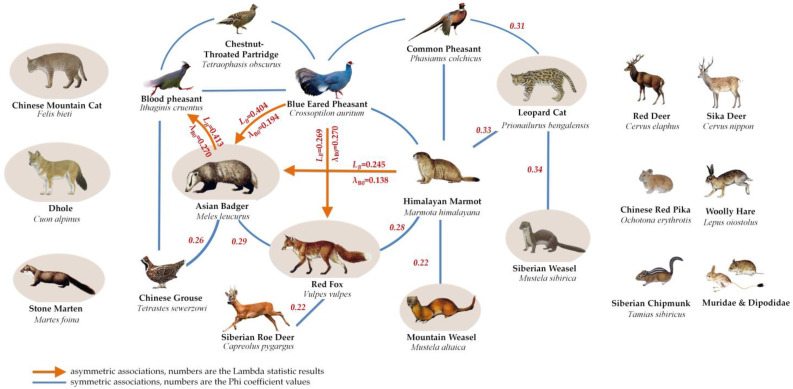
Spatial association network of terrestrial birds and mammals in LNNR.

According to the results of the phi coefficient (Figure 3), 12 out of the 20 terrestrial species recorded in the LNNR were connected in a spatial association network (Figure 4). Eight species, Chinese mountain cat, Dhole, stone marten, red deer, sika deer, Chinese red pika, woolly hare and Siberian chipmunk, had no significant spatial associations with other species and were not included in this network. The medium and small carnivores in the spatial network included the red fox, Asian badger, leopard cat, Siberian weasel and mountain weasel, accounting for 42% of the total species in the network. Medium and small carnivores comprised 12 pairs of positive spatial associations with other species in the network, accounting for 63% of the total number of species pairs. These pairs included the Asian badger and blue eared pheasant (*r_ø_* = 0.49, χ^2^ = 25.14, *p* < 0.001), Asian badger and Himalayan marmot (*r_ø_* = 0.38, χ^2^ = 14.66, *p* < 0.001), Asian badger and blood pheasant (*r_ø_* = 0.37, χ^2^ = 13.34, *p* < 0.001), leopard cat and Siberian weasel (*r_ø_* = 0.34, χ^2^ = 6.66, *p* < 0.05), leopard cat and Himalayan marmot (*r_ø_* = 0.33, χ^2^ = 10.60, *p* < 0.05), red fox and blue eared pheasant (*r_ø_* = 0.32, χ^2^ = 9.88, *p* < 0.05), leopard cat and common pheasant (*r_ø_* = 0.31, χ^2^ = 8.40, *p* < 0.05), red fox and Asian badger (*r_ø_* = 0.29, χ^2^ = 7.95, *p* < 0.05), red fox and Himalayan marmot (*r_ø_* = 0.28, χ^2^ = 7.45, *p* < 0.05), Asian badger and Chinese grouse (*r_ø_* = 0.26, χ^2^ = 6.20, *p* < 0.05), red fox and Siberian roe deer (*r_ø_* = 0.22, χ^2^ = 4.31, *p* < 0.05) and Himalayan marmot and mountain weasel (*r_ø_* = 0.22, χ^2^ = 4.12, *p* < 0.05).

### 3.3. Characterization of Spatial Associations of Medium and Small Carnivores

The lambda statistic was calculated for the species associated with medium and small carnivores in the LNNR. The following results were obtained:

Asymmetric association-biased benefits are observed between the red fox and blue eared pheasant, Asian badger and blue eared pheasant, Asian badger and Himalayan marmot and Asian badger and blood pheasant. The values range from 19.4–40.4% (*L_B_* = 0.404, λ_B0_ = 0.194, *p* ≤ 0.05) for Asian badger as a predictor of the blue eared pheasant; 13.8–34.5% (*L_B_* = 0.345, λ_B0_ = 0.138, *p* ≤ 0.05) for the Asian badger as a predictor of the Himalayan marmot; 10.2–26.9% (*L_B_* = 0.269, λ_B0_ = 0.102, *p* ≤ 0.05) for the red fox as a predictor of the blue eared pheasant and 6.1–23.1% (*L_B_* = 0.213, λ_B0_ = 0.061, *p* ≤ 0.05) for the blood pheasant as a predictor of the Asian badger (Figure 4);

Symmetric associations–competitive relations are observed between the leopard cat and Siberian weasel, leopard cat and Himalayan marmot, leopard cat and common pheasant, red fox and Asian badger, red fox and Himalayan marmot, Asian badger and Chinese grouse, red fox and Siberian roe deer and Himalayan marmot and mountain weasel.

### 3.4. Contribution of Medium and Small Carnivores to Network Maintenance

The spatial association network in the LNNR showed an average Shannon–Wiener index of 0.94. The *H*′ values of seven species were >0.94, including Himalayan marmot, blue eared pheasant, Asian badger, red fox, blood pheasant, leopard cat and common pheasant, which were the core species of the network. Five species with *H*′ values < 0.94 were considered peripheral species in the network, including chestnut-throated partridge, Chinese grouse, Siberian roe deer, mountain weasel and Siberian weasel (Table 3).

The contributions of medium and small carnivores to network maintenance were ordered as follows: Asian badger (*H′* = 1.584, 3rd); red fox (*H′* = 1.379, 4th); leopard cat (*H′* = 1.097, 6th); mountain weasel (*H′* = 0, 11th) and Siberian weasel (*H′* = 0, 11th). Therefore, the Asian badger, red fox and leopard cat played significant roles in network maintenance, which showed a high dependence on the network and were strongly restricted by the network. In contrast, the mountain weasel and Siberian weasel contributed the least to the network with a low dependence and weak restriction. The accumulative contribution of medium and small carnivores to the network maintenance was 35.8%.

## 4. Discussion

### 4.1. Status of Species Diversity in the LNNR

Our research results showed that Siberian roe deer, red fox, blue eared pheasant, Himalayan marmot and Asian badger occupied more than 30% of the camera traps and were distributed in all vegetation types and a large range of elevation, indicating that they were widely distributed in the LNNR.

In the 1960s, brown bear, gray wolf and other large carnivores used to live in the LNNR [21]. However, they were not detected in 2017 and 2018 [18]. Although the area of this study was larger than the last one, there was still no trace of brown bear and gray wolf; that means these species have very low population densities in the reserve.

According to the 2017–2020 investigation conducted by Xue et al. [22] and Hu et al. [23], brown bear, gray wolf, Chinese mountain cat and Dhole were distributed 200–300 km away from the LNNR, in the western part of the Qilian Mountain National Reserve (QNNR). However, they were not recorded in the eastern part of the QNNR which borders the LNNR. This suggests that the population density of brown bear, gray wolf, Dhole and Chinese mountain cat around the reserve is low or has even disappeared. Studies have shown that Dhole and Chinese mountain cat are mainly distributed in open desert habitats [24,25], and the LNNR is not a major distribution area for them. In addition, they are endangered species themselves; this may also be the reason why there are fewer detectedtraps and independent detections.

We also found that there were fewer detected traps and independent detections for Siberian Chipmunk and Siberian Weasel in this study. Relevant ecological studies have shown that they are widely distributed in broadleaf forest, coniferous forest, coniferous forest, shrub and places of human disturbance [26,27]. Although these vegetation types exist in the LNNR, their populations are still small.

### 4.2. Medium and Small Carnivores Cannot Replace the Ecological Roles of Large Carnivores

Carnivores of all sizes play an important role in regulating ecosystems [4,8,28,29]. Large carnivores are usually located at the top of the food web, limiting the population of local herbivores through predation and having control over medium and small carnivores [30,31]. The gray wolf, Dhole, Eurasian lynx and brown bear can prey on herbivores, such as deer, marmot and pika [32,33,34,35,36,37,38], but their population in the LNNR and surrounding areas are very small, or may have even disappeared, and they are unable to exert the role of population control on these herbivores.

Leopard cat [39], Chinese mountain cat [40], mountain weasel [41] and Siberian weasel [42] in the reserve mainly feed on mice, pika and birds. Although red fox can graze on ungulate corpses, they mainly prey on rodents and rabbits [43]. It can be observed that the reserve lacks predators that can put predation pressure on Siberian roe deer, sika deer and red deer. The fact that red deer and sika deer are not related to other species is well-illustrated in the spatial association network. Although the Siberian roe deer was located in the association network, it has a symmetrical spatial association with red fox, indicating that there is no significant favoritism between them, so the predation relationship is excluded. A similar pattern was observed among the Himalayan marmot, red fox, mountain weasel and leopard cat, suggesting no significant predation relationship exists between them either.

The above analysis shows that, in the absence of large carnivores, the existing medium and small carnivores cannot replace their ecological role that effectively control the population of large and medium-sized ungulates and large rodents.

### 4.3. Ecological Role of Medium and Small Carnivores

In the network, there were 12 pairs of associated species of 5 medium and small carnivores, accounting for 63% of the pairs of the total associated species. The Shannon–Weiner index calculation results show that the Asian badger, red fox and leopard cat were the core species of the network, and the overall contribution rate of the five species to the complexity of the network is 35.80%. These results indicate that medium and small carnivores play an important role in the maintenance of the network.

Some studies have shown that in the biasedly benefiting relationship, the benefited species tend to follow the beneficial species, forming cohesion, which is beneficial to the aggregation of species in the network (Wang et al., in press). Therefore, ecological interests tend to cause them to link and maintain ecological relationships in their spatial distributions. In the network, blood pheasant predicted Asian badger, Asian badger predicted the blue eared pheasant and Himalayan marmot and red fox predicted the blue eared pheasant, which formed a favorable ecological relationship between them. According to the above analysis, the existence of the blood pheasant can promote the survival of the Asian badger, and the existence of the Asian badger and red fox is conducive to the blue eared pheasant and Himalayan marmot finding a suitable habitat and food resources. Thus, it can be observed that the Asian badger and red fox act as aggregators of other species in the network.

Interestingly, except for the relationship that the blood pheasant attracted the Asian badger, the direction of the other three pairs of unidirectional asymmetric relationships were the opposite: carnivores attracted other species, showing no significant predation relationship (Figure 4). Without the restriction of large carnivores, the medium and small carnivores are less difficult to feed, which would make their predation of specific species reduced and the predation more random [7,44,45,46]. Similarly, the leopard cat, Siberian weasel and mountain weasel presented symmetrical spatial associations with other species, showing greater competition than predation.

The analysis presented above suggests that in the absence of large carnivores, the predation of medium and small carnivores is more randomized, leading to the diffusion of the intensity of interaction between them and their prey to more pathways [44,45,46]; thus, the single predation relationship was no longer so significant. This was probably the main reason why the Chinese red pika, woolly hare, Siberian chipmunk and “Muridae and Dipodidae” did not enter the network.

### 4.4. Community Development Trends

The example of Scottish red deer shows that ungulates, in the absence of predation control, can cause habitat damage that can affect the survival of other species [47]. This study showed that the population of Siberian roe deer, red deer and sika deer in the LNNR lacks predation control, which should be addressed by the managers. In the future, if the population of red deer and sika deer continue to increase, the intensity of the competition with Siberian roe deer may increase, which will exert a limiting effect on the population development of Siberian roe deer.

Other studies have shown that parasite infection can lead to a large number of animal deaths [48], and the transmission of diseases between species will also affect the population health of species, for example, the rabies virus can be transmitted between red fox and domestic dogs [49,50], and plagues will spread among Himalayan marmot, red fox, Asian badger, red deer, stone marten and other species [51]. With the increase in the population of medium carnivores and herbivores in the reserve, the spread of diseases and parasites will also be enhanced. When the disease and parasite infection rates reach a high level, this may become one of the factors restricting the population growth of these species.

The status of the network reflects the changes in community composition and structure after the decline in large carnivores and other species, and the above predictions reflect the possible trend of development of the community. Due to the short duration of this survey, a long-term monitoring is needed to accurately comprehend the development trend of the community and the dynamic changes of the populations.

## 5. Conclusions

Our study constructed a spatial association network that reflects the community status and found that medium and small carnivores play an important ecological role in the maintenance of community, and they cannot replace the ecological roles of large carnivores. This study provided up-to-date information for managers to understand community development trends and would be a guide to the direction and focus of conservation efforts.

## Figures and Tables

**Table 1:** Contingency table of the phi coefficients.

Species Y	Species X		
	Presence	Absence	Total
Presence	A	B	A + B
Absence	C	D	C + D
Total	A + C	B + D	N

**Table 2:** Terrestrial birds and mammals detected in LNNR.

Orde	Family	Species
Carnivora	Canidae	Red Fox (*Vulpes vulpes*)
		Dhole (*Cuon alpinus*)
	Mustelidae	Siberian Weasel (*Mustela sibirica*)
		Mountain Weasel (*Mustela altaica*)
		Asian Badger (*Meles leucurus*)
		Stone Marten (*Martes foina*)
	Felidae	Chinese Mountain Cat (*Felis bieti*)
		Leopard Cat (*Prionailurus bengalensis*)
Cetartiodactyla	Cervidae	Sike Deer (*Cervus nippon*)
		Red Deer (*Cervus elaphus*)
		Siberian Roe Deer (*Capreolus pygargus*)
Rodentia	Sciuridae	Siberian Chipmunk (*Tamias sibiricus*)
		Himalayan Marmot (*Marmota himalayana*)
		Muridae and Dipodidae
Lagomorpha	Ochotonidae	Chinese Red Pika (*Ochotona erythrotis*)
	Leporidae	Woolly Hare (*Lepus oiostolus*)
Galliformes	Phasianidae	Chinese Grouse (*Tetrastes sewerzowi*)
		Blue Eared Pheasant (*Crossoptilon auritum*)
		Blood Pheasant (*Ithaginis cruentus*)
		Chestnut-throated Partridge (*Tetraophasis obscurus*)
		Common Pheasant (*Phasianus colchicus*)

**Table 3:** Contribution of species to network complexity.

Species	Shannon–Wiener Index *(H′*)	Contribution to Network Complexity
Himalayan marmot *	1.770	15.62%
Blue eared pheasant *	1.757	15.51%
Asian badger *	1.584	13.98%
Red fox *	1.379	12.17%
Blood pheasant *	1.334	11.78%
Leopard cat *	1.097	9.69%
Common pheasant *	1.087	9.59%
Chestnut-throated partridge	0.693	6.12%
Chinese grouse	0.628	5.54%
Siberian roe deer	0.000	0.00%
Mountain weasel	0.000	0.00%
Siberian weasel	0.000	0.00%

Note: species with * are the core species in the network.

## Data Availability

The data presented in this study are available from the corresponding author on reasonable request.
